# A Soft-Pruning Method Applied During Training of Spiking Neural Networks for In-memory Computing Applications

**DOI:** 10.3389/fnins.2019.00405

**Published:** 2019-04-26

**Authors:** Yuhan Shi, Leon Nguyen, Sangheon Oh, Xin Liu, Duygu Kuzum

**Affiliations:** Electrical and Computer Engineering Department, University of California, San Diego, San Diego, CA, United States

**Keywords:** spiking neural networks, unsupervised learning, handwriting recognition, pruning, in-memory computing, emerging non-volatile memory

## Abstract

Inspired from the computational efficiency of the biological brain, spiking neural networks (SNNs) emulate biological neural networks, neural codes, dynamics, and circuitry. SNNs show great potential for the implementation of unsupervised learning using in-memory computing. Here, we report an algorithmic optimization that improves energy efficiency of online learning with SNNs on emerging non-volatile memory (eNVM) devices. We develop a pruning method for SNNs by exploiting the output firing characteristics of neurons. Our pruning method can be applied during network training, which is different from previous approaches in the literature that employ pruning on already-trained networks. This approach prevents unnecessary updates of network parameters during training. This algorithmic optimization can complement the energy efficiency of eNVM technology, which offers a unique in-memory computing platform for the parallelization of neural network operations. Our SNN maintains ~90% classification accuracy on the MNIST dataset with up to ~75% pruning, significantly reducing the number of weight updates. The SNN and pruning scheme developed in this work can pave the way toward applications of eNVM based neuro-inspired systems for energy efficient online learning in low power applications.

## Introduction

In recent years, brain-inspired spiking neural networks (SNNs) have been attracting significant attention due to their computational advantages. SNNs allow sparse and event-driven parameter updates during network training (Maass, 1997; Nessler et al., 2013; Tavanaei et al., 2016; Kulkarni and Rajendran, 2018). This results in lower energy consumption, which is appealing for hardware implementations (Cruz-Albrecht et al., 2012; Merolla et al., 2014; Neftci et al., 2014; Cao et al., 2015). Emerging non-volatile memory (eNVM) arrays have been proposed as a promising in-memory computing platform to implement SNN training in an energy efficient manner. eNVM devices can implement spike-timing-dependent plasticity (STDP) (Jo et al., 2010; Kuzum et al., 2011), which is a commonly used weight update rule in SNNs. Most demonstrations utilize eNVM crossbar arrays to parallelize computation of the inner product (Alibart et al., 2013; Choi et al., 2015; Prezioso et al., 2015; Eryilmaz et al., 2016; Ge et al., 2017; Wong, 2018). In addition, there are several works focus on using eNVM hardware such as spintronic devices or crossbars with additional algorithmic optimization of STDP learning rules to perform hardware implementation of SNN (Sengupta et al., 2016; Srinivasan et al., 2016; Ankit et al., 2017; Panda et al., 2017a,b). While eNVM crossbar arrays improve energy efficiency at a device level for SNN training, network level algorithmic optimization is still important to further improve energy efficiency for wide adoption of SNNs in low power applications.

Pruning network parameters, i.e., synaptic weights, is a recent algorithmic optimization (Han et al., 2015) that is widely used for compressing the network to improve the energy efficiency for the inference operation of deep neural networks. Although synaptic pruning has been demonstrated in many biophysical SNN models (Iglesias and Villa, 2007; Deger et al., 2012, 2017; Kappel et al., 2015; Spiess et al., 2016), how the pruning can be used for non-biophysical SNN has not been fully explored yet. Moreover, this method is applied on already-trained networks and it does not address the high-energy consumption during training, which requires iterative weight updates. A new approach toward network training that improves the energy efficiency of SNNs is crucial to develop online learning systems that can learn and perform inference in real world scenarios.

Here, we develop an algorithm to prune during training for SNNs with eNVMs to improve network level energy efficiency for in-memory computing applications. Although Rathi et al. (Rathi et al., 2018) has showed pruning in SNN before, there are several key innovations and differences of the pruning method in this work compared to Rathi et al.' work. Our method considers the spiking activity of the output neurons to decide when to prune during the training while Rathi et al. performs the pruning at regular intervals for every batch without considering the characteristics of the output neurons. In addition, once the weights have been pruned during the training, we do not update the pruned weights for the rest of the training while Rathi et al. only temporally removes the pruned weights and they can still be updated when new batches present to the network. Finally, we develop soft-pruning as an extension of pruning. Soft-pruning sets the pruned weights to a constant non-zero values. Therefore, it is novel in terms of treating pruned weights. Rathi et al. only implement pruning.

Our paper is organized as follows: first, we describe our unsupervised SNN model and the weight update rule. Then, we introduce a pruning method that exploits spiking characteristics of the SNN to decrease the number of weight updates and thus energy consumption during training. Finally, we discuss how our SNN training and pruning algorithm can potentially be realized using eNVM crossbar arrays and perform circuit-level simulations to confirm the feasibility for online unsupervised learning to reduce the energy consumption and training time.

In section Input layer to section Testing, we discuss our SNN model and the algorithms relating to weight updates. In section Pruning during training, we discuss methods to prune during training. In section Results and discussion, we discuss our software simulation results, compare our SNN with state-of-the-art unsupervised SNN algorithms on MNIST and explore the method to implement our SNN model and pruning algorithm using the eNVM crossbar array through circuit-level simulations.

## Neural Network Architecture

Inspired by the information transfer in biological neurons via precise spike timing, SNNs temporally encode the inputs and outputs of a neural network layer using spike trains. The weights of the SNN are updated via a biologically plausible STDP, which modulates weights based on the timing of input and output spikes (Nessler et al., 2013; Tavanaei et al., 2016). This can be easily implemented on an eNVM crossbar array (Kuzum et al., 2011), making it ideal for online learning in hardware.

**Figure 1:**
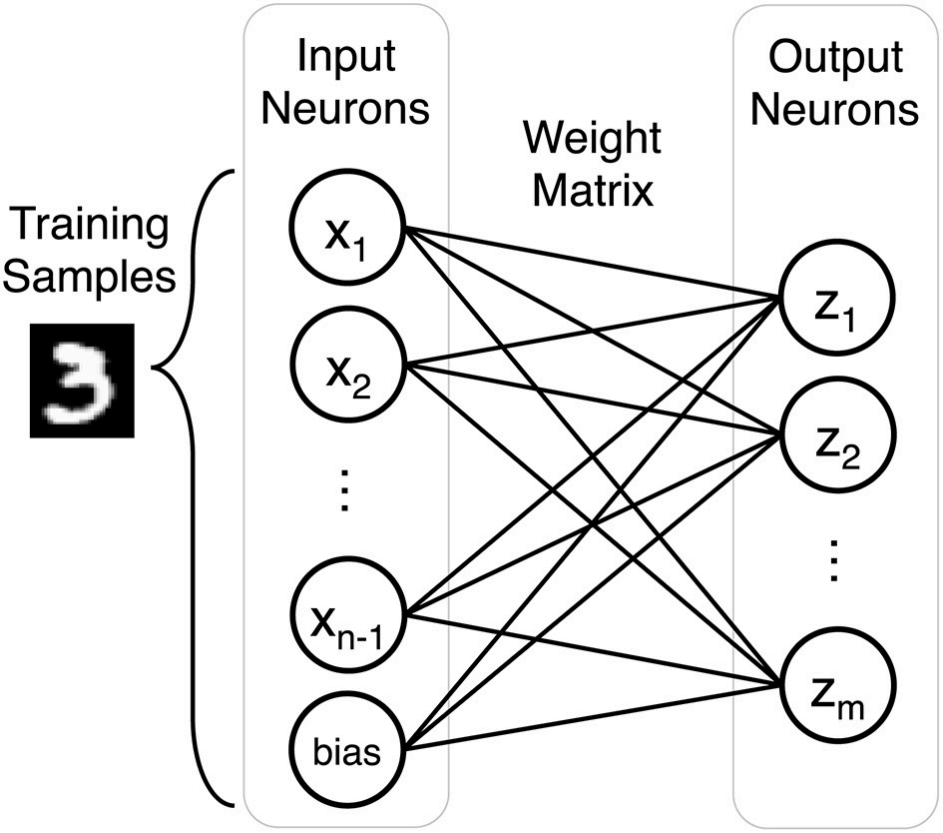
Each training sample is represented by *n* input neurons (*n*-1 input neurons and a bias term). The weight matrix is an *m* by *n* array composed of the weights that connect input neurons to output neurons in the single layer network.

Our SNN performs unsupervised classification of handwritten digits from the MNIST dataset. It is a single layer network defined by the number of inputs neurons *n*, the number of outputs neurons *m*, and an *m* by *n* weight matrix. The number of input neurons can vary depending on preprocessing, but by default there are 784 input neurons to account for each grayscale pixel in a training sample. The output layer consists of 500 neurons to classify the 10 classes of the MNIST dataset (60,000 training images and 10,000 testing images). Figure 1 describes the fully connected network architecture.

As an overview of the pipeline, we first train the SNN by sequentially presenting samples from the training set. The purpose of training is to develop the weights of each output neuron so that they selectively fire for a certain class in MNIST. Afterwards, we present the training set for a second time to label each trained output neuron with the class of training samples that has the highest mean firing rate. This organizes the output neurons into populations that each respond to one of the classes. Finally, we test the SNN by predicting the label of each of the test samples based the class of output neurons with the highest mean firing rate.

### Input Layer

We first remove the pixels that are used to represent the background in at least 95% of the training samples to reduce the number of input layer neurons. Because the grayscale pixels have intensity values in the range [0, 1], the pixels with a value of 0 correspond to the background and are thus checked for removal. After this step, we retain 397 of the original 784 pixels, reducing the complexity of the SNN. Therefore, we have 398 input neurons for a given training sample after accounting for an additional bias input neuron, which has a value of 1. Our output neurons do not have refractory periods and there is no lateral inhibition between them.

**Figure 2:**
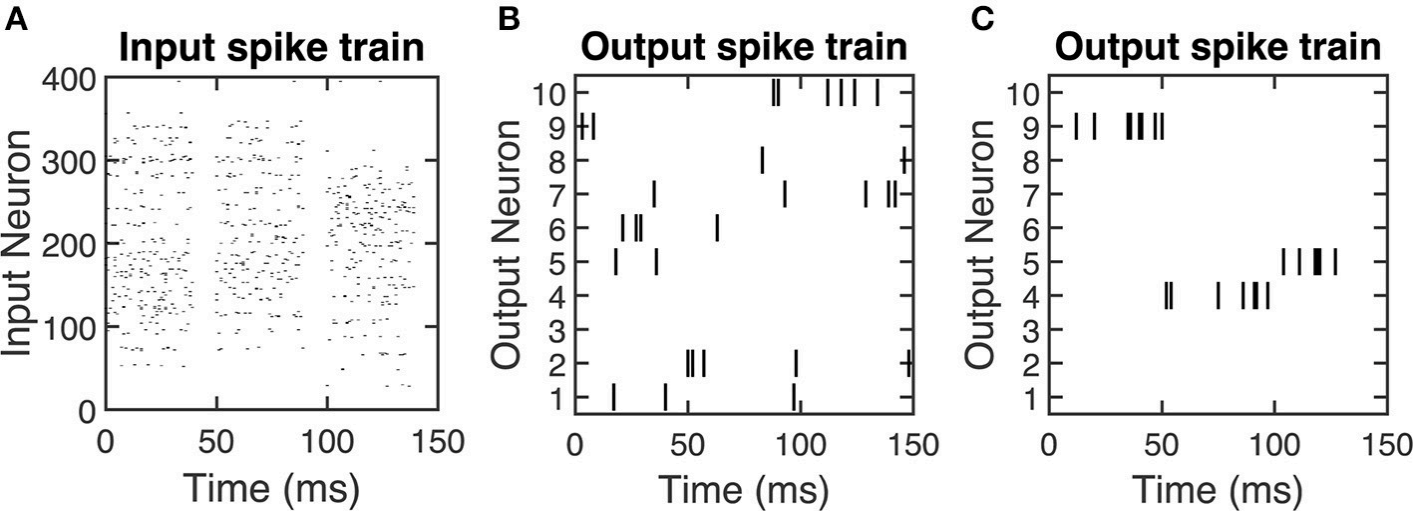
Spike raster plots showing examples of **(A)** input spiking activity, **(B)** output spiking activity for an untrained SNN, and **(C)** output spiking activity for a trained SNN. For **(B)** and **(C)**, 10 output neurons' spiking activities are selected as a representative example. After the SNN is trained, the output spike firing activity is more coordinated, which is indicated by the output neurons selectively firing to certain input stimuli. The time duration on the *x* axis indicates the presentation of training samples. Since the output neuron firing rate is 200 Hz, therefore there are around 10 spikes (# of spikes = presentation time × frequency = 50ms × 0.001 × 200 Hz = 10) will be generated within 50 ms presentation time.

We encode each of these inputs as a Poisson spike train at a frequency of 200 times its value, leading to a maximum input firing rate of 200 Hz. We round the timing of each spike that is generated by the Poisson process to the nearest millisecond, which is the time of one time step in the SNN. The SNN displays each training sample for the first 40 ms of a 50 ms presentation period, and thus the input spikes for a given training sample can only occur in this 40 ms window. Figure 2A shows an example of the input spiking activity for the duration of three training samples.

### Output Layer

For output spikes, we use the Bayesian winner-take-all (WTA) firing model (Nessler et al., 2013). Unlike traditional integrate-and-fire models (Gupta and Long, 2007; Diehl and Cook, 2015a), this model is shown to demonstrate Bayes' rule (Nessler et al., 2013), which is a probabilistic model for learning and cognitive development (Perfors et al., 2011). The SNN fires an output spike from any given output neuron according to a 200 Hz Poisson process. The output neuron that fires is chosen from a softmax distribution of the output neurons' membrane potentials:

(1) $$p\left(u_{k}\right)=\frac{\exp\left(u_{k}\right)}{\sum_{i=1}^{m}\exp\left(u_{i}\right)},$$

where {p(_*u*_*k*_)}*k* = 1, …, *m*_ is the softmax probability distribution of the membrane potentials {_*u*_*k*_}*k* = 1, …, *m*_. *m* is the number of output neurons. Our firing mechanism is probabilistic instead of hard thresholding the membrane potentials. Therefore, the neuron with higher membrane potential means that it has higher chance to fire. We calculate membrane potentials *u*_*k*_ using (2)

(2) $$u_{k}=\;\sum_{i}W_{ki}X_{i}+b_{k}$$

where *W*_*ki*_ is the weight between input neuron *i* and output neuron *k, X*_*i*_ is the spike train generated by input neuron *i* and *b*_*k*_ is the weight of the bias term. Equation (2) calculates an output neuron's membrane potential as the inner product between the input spikes at a given time step and the output neuron's weights, but this does not need to be integrated with each time step. Instead, we only calculate the membrane potentials at time steps when an output neuron fires because it is only used to determine which output neuron to fire. This removes additional parameters and resources needed with typical integrate-and-fire neuron models, which use the membrane potential to also find when to fire output neurons, allowing for a more efficient hardware implementation.

### Weight Updates: STDP Rule

**Figure 3:**
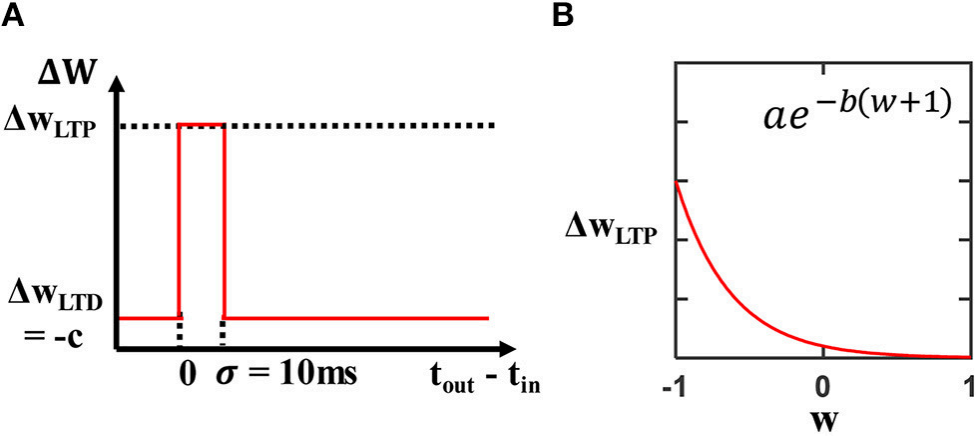
**(A)** STDP rule showing the 10 ms window for an output-input spike time difference (*t*_*out*_–*t*_*in*_) that determines whether an LTP or an LTD update is performed. If the output-input spike time difference (*t*_*out*_–*t*_*in*_) is within 10 ms, the weight corresponding to this input-output synapse is updated via LTP. Otherwise, the weight is updated via LTD. The LTP update is an exponential function that depends on the current weight, and the LTD update is a constant. **(B)** The exponential LTP update is dependent on the current weight *w* and it helps keep the weight values within the range [−1, 1].

When an output neuron fires, a simple STDP rule determines which weights to update via long-term potentiation (LTP) or long-term depression (LTD). As shown in Figure 3A, if an input neuron's most recent spike is within σ = 10 ms of the output spike, then the weight for this input-output synapse is increased (LTP). Otherwise, if it is beyond this 10 ms window of the output spike, then the weight is decreased (LTD).

This 10 ms window is in accordance with the fact that training samples are not displayed during the final 10 ms of their presentation period—they are only displayed for the first 40 ms of the 50 ms presentation period. Thus, there are no input spikes in the final 10 ms of each presentation, as seen in Figure 2A. Therefore, this STDP window prevents LTP weight updates that are potentially caused by the input spiking activity of the previous training sample. For example, when a new training sample is inputted to the SNN, an output spike occurring at simulation time *t* = 50 ms cannot have a spike-timing difference with an input spike occurring from *t* = 41 ms to *t* = 49 ms, since this is within the 10 ms window for LTP weight updates.

Figure 2B shows an example of the output spiking activity for 10 representative output neurons with randomly initialized weights, illustrating the random spiking activity of an untrained SNN. The effect of performing weight updates is to train the network to selectively fire to certain classes of inputs. At the start of training, we randomly initialize all weight values between [−1, 1], and the LTP and LTD update rules keep the weight values within the range [−1, 1]. The LTP weight update is an exponential function of the form Δ$w_{LTP}\left(w\right)=ae^{-b\left(w+1\right)}$ (Figure 3B), where *a* ∈ {ℝ:0 < *a* < 1} and *b* ∈ ℝ_>0_ are parameters that control the scale of the exponential, and *w* is the current weight value. For LTP updates to keep weight values within the upper bound of 1, we pick the parameters such that the weight update decays toward 0 as the current weight approaches 1. As a result, exponential LTP updates will guarantee that the weights converge to the upper bound of 1.

Unlike LTP, the LTD weight update is a constant function that disregards the current weight value: *w*_*LTD*_ = −*c*, where *c* ∈ {ℝ:0 < *c* < 1} is a parameter that controls the magnitude of the weight decrease. Because there is no guarantee of convergence as with the exponential LTP update, the SNN clips weights to the lower bound of −1. Alternatively, we can have an exponential LTD update that is mirrored about *w* = 0 from the exponential LTP update, i.e., Δ$w_{LTD}\left(w\right)=-ae^{b\left(w-1\right)}$, and choose parameters to have weight convergence as in the case of LTP. However, the constant LTD update is easier to implement in hardware since there are less parameters to tune. The specific parameter choices of *a, b* ,and *c* are shown in Table 1 and they come from cross validation of the parameter set to optimize the classification accuracy. Several previously published papers have proposed probabilistic synapses to perform STDP weight update (Vincent et al., 2014; Srinivasan et al., 2016). It is worth to note that the synapses in our network is deterministic and only the firing mechanism of output neurons is probabilistic as explained in section Output layer.

**Table 1:** Simulation parameters used in training, labeling and testing for this work.

**Parameters**		**10–digits**		
		**Training**	**Labeling**	**Testing**
# of neuron	Input	398		
	Output	500		
Firing rate (Hz)	Input	200	200	200
	Output	200	200	600
Image presenting time (ms)		50	50	200
Neuron removal threshold		–	0.75	–
Pruning threshold	Prune parameter *(r)*	10	–	–
	Spice count	8	–	–
STDP		a = 0.0667 b = 2.5 c = 0.0167		

### Scaling Weight Updates as a Normalization Method

**Figure 4:**
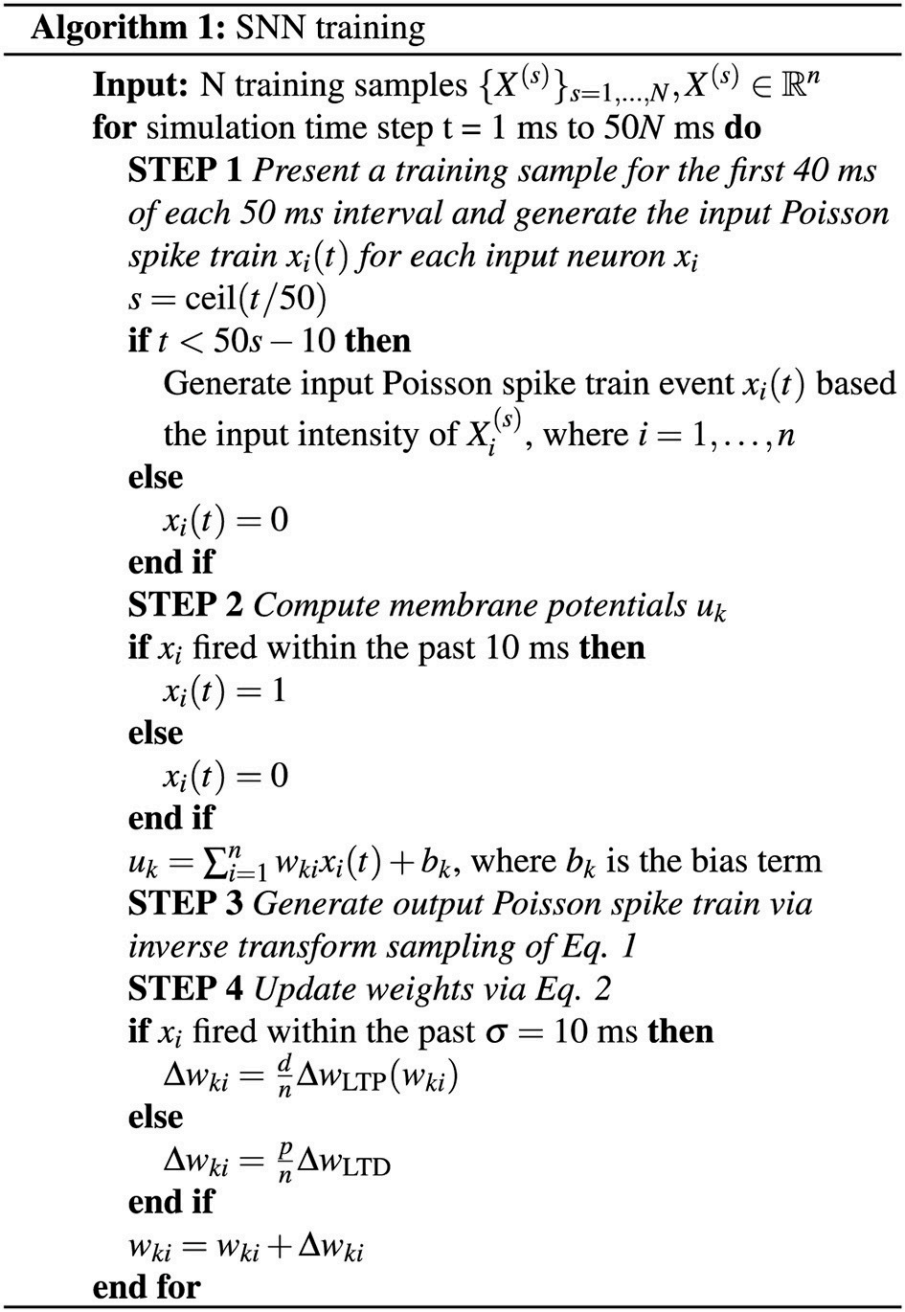
SNN training algorithm.

To perform a weight update, we add to the current weight *w*_*t*_ the weight update, which is scaled by an additional factor depending on whether the update is LTP or LTD:

(3) $$w_{t+1}=\{\begin{array}{c} w_{t}+\frac{d}{n}\Delta w_{LTP}(w_{t}),\;LTP\\ w_{t}+\frac{p}{n}\Delta w_{LTD},\;LTD \end{array}$$

where *d* is the number of weights to undergo LTD, *p* is the number of weights to undergo LTP, and *n* is the total number of weights for an output neuron, which also corresponds to the number of input neurons. Because of the STDP rule, all *n* weights of an output neuron are updated at any given output neuron firing event, which means that *d* + *p* = *n*. Because the number of LTP updates is often disproportionate with that of LTD due to the probabilistic spike firing, the scaling factors *d* and *p* keep the net weight change of both types of updates proportional so that for all output neurons, the distribution of weight values have roughly the same mean and variance. With this, an overview of the SNN training method is outlined in Figure 4.

This scaling of LTP and LTD weight updates is used to prevent certain output neurons from firing more than others. It effectively normalizes the weight distributions of each output neuron so that they fire according to the correlation between their weights and the training sample, rather than firing because the magnitude of their weights artificially increases their membrane potential. This foregoes the need to normalize the weight distributions of each output neuron through calculating the mean and standard deviation, which requires additional resources when implementing the weight update in hardware.

### Testing

After training is done, we fix the trained weights and assign a class to each neuron by the following steps: First, we present the whole training set to the SNN and record the cumulative number of output spikes *N*_*kj*_, where *k* = 1, …, *m* (*m* is number of output neurons) and *j* = 1, …, *n* (*n* is number of classes, for MNIST, *n* = 10). Then, for each output neuron *i*, we calculate its response probability *Z*_*kj*_ to each class *j* using Eq. (4). Finally, each neuron k is assigned to the class that gives the highest response probability *Z*_*kj*_.

(4) $$Z_{kj}=\;\frac{N_{kj}}{\sum_{j=1}^{n}N_{kj}}$$

After training and labeling are done, we fix the weights and present test set to our network. We use Eq. (5) to predict the class of each sample, where *S*_*jk*_ is the number of spikes for the kth output neuron that are labeled as class *j* and *N*_*j*_ is the number of output neurons labeled as class *j*.

(5) $$J=\underset{j}{argmax}\frac{\sum_{k=1}^{N_{j}}S_{jk}}{N_{j}}$$

### Pruning During Training

Pruning is a concept in machine learning that removes redundant branches from a decision tree to reduce complexity and improve accuracy of the classifier. It prevents overfitting by learning the general structure of the input data instead of learning minute details. Han et al. implement pruning on trained convolutional neural networks to remove unimportant weights that have low contribution to the output (Han et al., 2015). For example, weights with values close to 0 can be removed since their inner product with their respective inputs will yield low output values. This removal effectively sets the weight values to 0, allowing for a sparser representation of the network for mobile applications while still retaining the same classification performance. Instead of pruning after training, we propose a method to prune during training on SNNs to reduce the number of weight updates.

Our implementation of pruning removes unimportant weights belonging to each output neuron, and each output neuron is only pruned once during training. When an output neuron fires, its weights can potentially be pruned based on the level of development in its weights. There is a tradeoff in choosing when to prune an output neuron. If we prune weights early during training, we save computation by not having to update these weights later on. However, by pruning early, the weights might not be trained enough to recognize a certain class in the dataset at the time of pruning, and this early pruning can hamper the future development of the weights. Conversely, pruning late better insures that the weights are trained at the expense of computing more weight updates.

**Figure 5:**
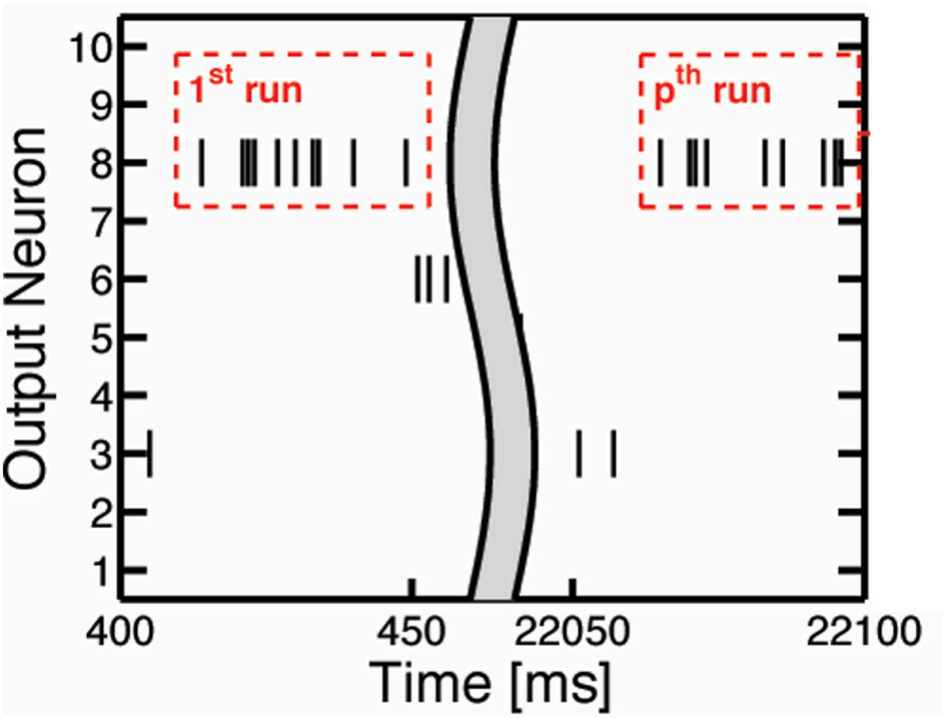
The illustration of consecutive output spikes of 10 output neurons as a representative example. The consecutive output spikes of Neuron 8 are boxed in red.

To determine when to prune the weights of an output neuron, we refer to the spiking activity of the output neurons. The output neuron spiking activity is an inherent feature of SNNs that indicates the level of development in an output neuron's weights. Once an output neuron is trained enough to recognize a certain class from the dataset, it will start to fire more consistently, as in Figure 2C, due to its high membrane potential. To quantify this consistent output neuron firing behavior, we accumulate a count of the occurrences where there are at least 8 consecutive output spikes (Table 1) from a specific output neuron during the 40 ms presentation period of a training sample. This count is kept for each output neuron as shown in Figure 5, and once an output neuron accumulates *r* (*r* = 10 in our case as shown in Table 1) such counts during training, the SNN prunes a user-defined percentage of its weights. We choose to look for 8 consecutive output spikes based on the 200 Hz output firing rate, and the 10 count threshold is a hyperparameter to control how early or late to prune an output neuron. It is worth noting that the pruning percentages are set externally in our method and they can be chosen according to the dataset, the accuracy requirement and power/latency budget of the specific applications.

**Figure 6:**
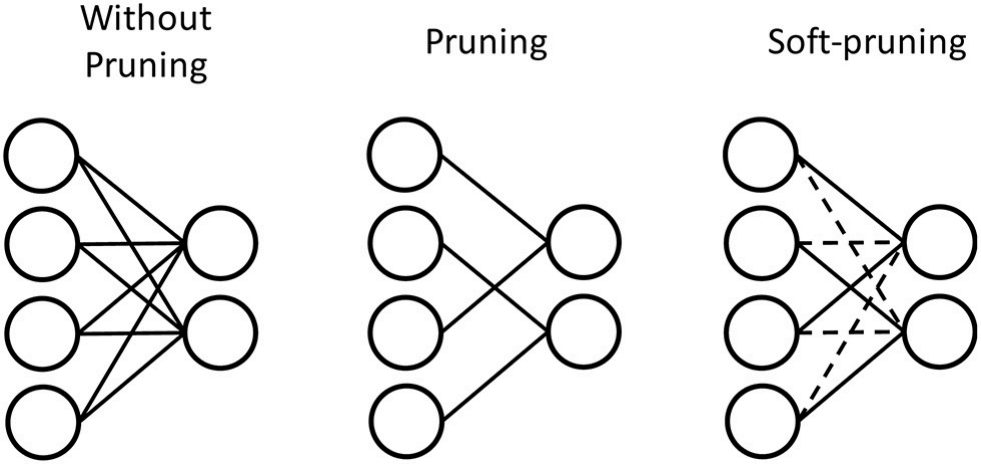
Pruning prunes weights with values around 0 by setting these weights to 0. Much like a sparse matrix, these weights do not have to be stored in memory if their index is stored. Therefore, the synapses are physically removed to represent pruning. In contrast, soft-pruning prunes weights with values meeting certain criteria by keeping these weights constant at a certain value for the rest of training. Therefore, the pruned weights still need to be stored because they can be nonzero, but they are represented by dashed lines to indicate that they no longer need to be updated during training of the SNN.

We explore two different methods of pruning in this work. We use the conventional pruning method (Han et al., 2015) to prune the weights by setting their values to 0, which we also refer to pruning in this work. We also investigate a soft-pruning method (Kijsirikul and Chongkasemwongse, 2001) as an extension of conventional pruning. Instead of completely removing the weights by setting them to 0, soft-pruning keeps the pruned weights constant at their current values for the remainder of training, or even keeping certain weights constant at the lowest or highest weight values allowed. This allows for more flexible criteria in regard to which weights are pruned, and what values they take as a result of pruning. In this work, we set the pruned weights to the lowest possible weight values, which is −1 for our network. The advantage of pruning is in reducing the representation of the weight matrix by introducing more sparsity. Figure 6 demonstrates this by the physical removal of synapses. However, depending on the dataset, the number of weights that will be close enough to 0 to comfortably prune without losing important information can vary. While soft-pruning does not necessarily introduce more sparsity, it can allow for more weights to be pruned, thus saving computation by preventing more weight updates without drastically altering the weight distribution. Figure 6 shows the pruned weights via soft-pruning as dashed lines to indicate that they still need to be stored in memory and participate in the testing. Soft-pruning does not increase the sparsity of weight matrix. However, since these weights are no longer updated, this can reduce energy consumption in the hardware implementation.

**Figure 7:**
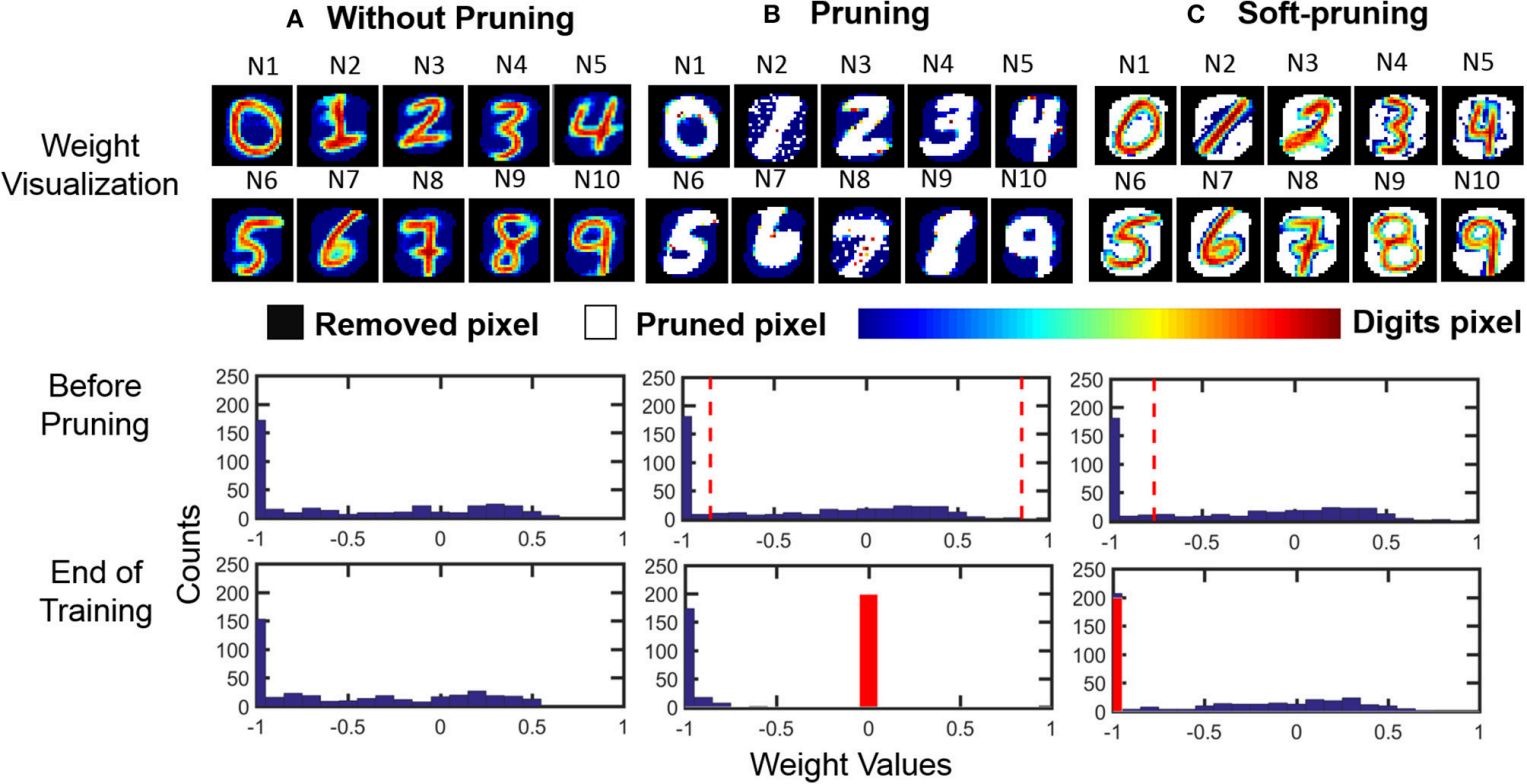
Pruning example. (Top row) Weight visualization of the 10 representative output neurons after the SNN is trained in 3 different cases: (1) without pruning, (2) with pruning (while pruning 50% of the weights), and (3) with soft-pruning (while pruning 50% of the weights). (Middle row) Weight distribution of a representative output neuron (N3) before it is about to be pruned during training. (Bottom row) Weight distribution of the same output neuron at the end of training. It is worth to note that we only recover the removed pixels (black pixels) for visualizing the learned weights. Since those pixels are not used during training, histograms only include corresponding digit pixels (color bar) and pruned pixels (white pixels). **(A)** Without pruning, the STDP rule causes the weights for the MNIST dataset to follow a distribution where many of the weight values saturate at the lowest possible value. These low weights represent the background of MNIST training samples that are being learned by the SNN. **(B)** Pruning prunes the weights between the two dashed lines, which represent the 50% of the weights that centered about 0, and sets their values to 0 (red bar). **(C)** Soft-pruning prunes the weights to the left of the dashed line, which represent the 50% of the weights that are the lowest-valued weights, to the lowest possible value, which is at −1 (red bar). For both pruning and soft-pruning, the weights that are not pruned continue to develop for the rest of training.

The usage of these two different pruning methods is dependent on the dataset to be classified. For example, the features of an image from MNIST can be separated into binary categories, i.e., the foreground and the background. In such a case, an example of soft-pruning is to prune a percentage of the lowest-valued weights of an output neuron by keeping these weight values at the lowest possible value, which for our SNN is −1. This variant of soft-pruning is analogous to learning a weight representation where the pixels representing the background take a single value, but the pixels representing the foreground can take on a range of values. Intuitively, soft-pruning results in a weight representation that does not waste resources to encode the black background pixels in MNIST in order to learn the details of the foreground, which can have varying levels of intensity due to the stroke weight of the handwriting. The top row of Figure 7 shows an example of the learned weight visualizations of 10 representative output neurons when the SNN is trained on the MNIST dataset in three cases: without pruning, with pruning, and with soft-pruning. By the seeding of the random number generator, we control the spiking activity of all three cases so that the third output neuron (N3) is the first to meet the pruning criteria. Therefore, up to the point before N3 is pruned, the SNNs for each of the three cases have the exact same spiking activity and weight update history for all output neurons. For example, the middle row of Figure 7 shows that N3's weight distribution is the same for all three cases. After this point, the different pruning methods between the three cases cause the weights of the output neurons between each case to develop differently.

Comparing the weight distributions for N3 in the final row of Figure 7, we can verify that soft-pruning is more reasonable than pruning for the MNIST dataset because it better preserves the shape of the original weight distribution, without pruning, in Figure 7A. In this example, we use both pruning methods to prune half of an output neuron's weights to clearly demonstrate the effect of each pruning method on the weight distribution. For pruning in Figure 7B, pruning 50% of the weights centered about the value 0 results in compressing a wide range of weights, shown by the space between the two dashed lines in the middle panel. Effectively, these pruned weights, most of which represent the foreground features of the MNIST dataset, are set to 0. Although the final panel of Figure 7B shows a somewhat binary weight distribution, which matches the binary foreground and background features of the MNIST dataset that we want to learn, the problem is that the shape of this weight distribution is drastically different than that of the weight distribution when the weights develop without pruning, as seen in the final panel of Figure 7A. In contrast, the effect of soft-pruning on the shape of the weight distribution, as seen in the final panel of Figure 7C, is minimal when compared to the case without pruning. Therefore, the pruned output neurons will produce comparable membrane potentials to the unpruned output neurons during training, resulting in balanced training between all output neurons.

With more complex datasets, e.g., color images, we might want to prune weights by setting weights around 0 to 0, or by setting weights to their current value. Han et al. demonstrate the former (Han et al., 2015). In the latter case, an interpretation can be that we set unimportant weights to their current value with the assumption that their current representation is already satisfactory for learning. Another approach is to freeze important, high-valued weights, which is a recently explored neuro-inspired concept called consolidation (Mnih et al., 2015).

## Results and Discussion

**Figure 8:**
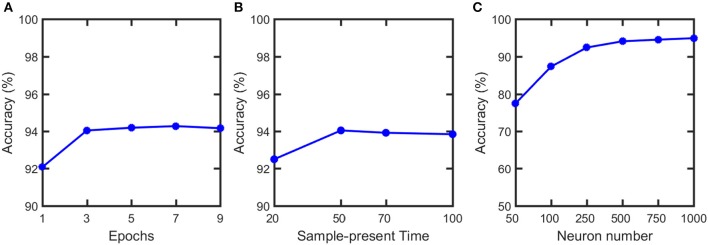
Classification accuracy vs. **(A)** number of training epochs, **(B)** sample-present time and **(C)** output neuron numbers. Classification accuracy does not have noticeable increase after 3 epochs and 50 ms present time. Therefore, 3 epochs and 50 ms are used in the training. Although the accuracy can be further improved if neuron number increases, it will significantly increase the simulation time. Therefore, we choose to use 500 output neurons in our simulation.

We simulate our SNN model, pruning and soft-pruning in MATLAB. To determine a suitable size for the training dataset, we find via Figure 8A that three epochs (60,000 training samples per epoch) is sufficient to reach ~94% classification accuracy. Additionally, from Figure 8B, we use a 50 ms presentation period per training sample because longer presentation times show diminishing improvements in classification accuracy. Figure 8C shows the accuracy increases as the number of output neurons increase. However, adding output neurons will significantly increase the simulation time. Therefore, we choose to use 500 output neurons.

**Figure 9:**
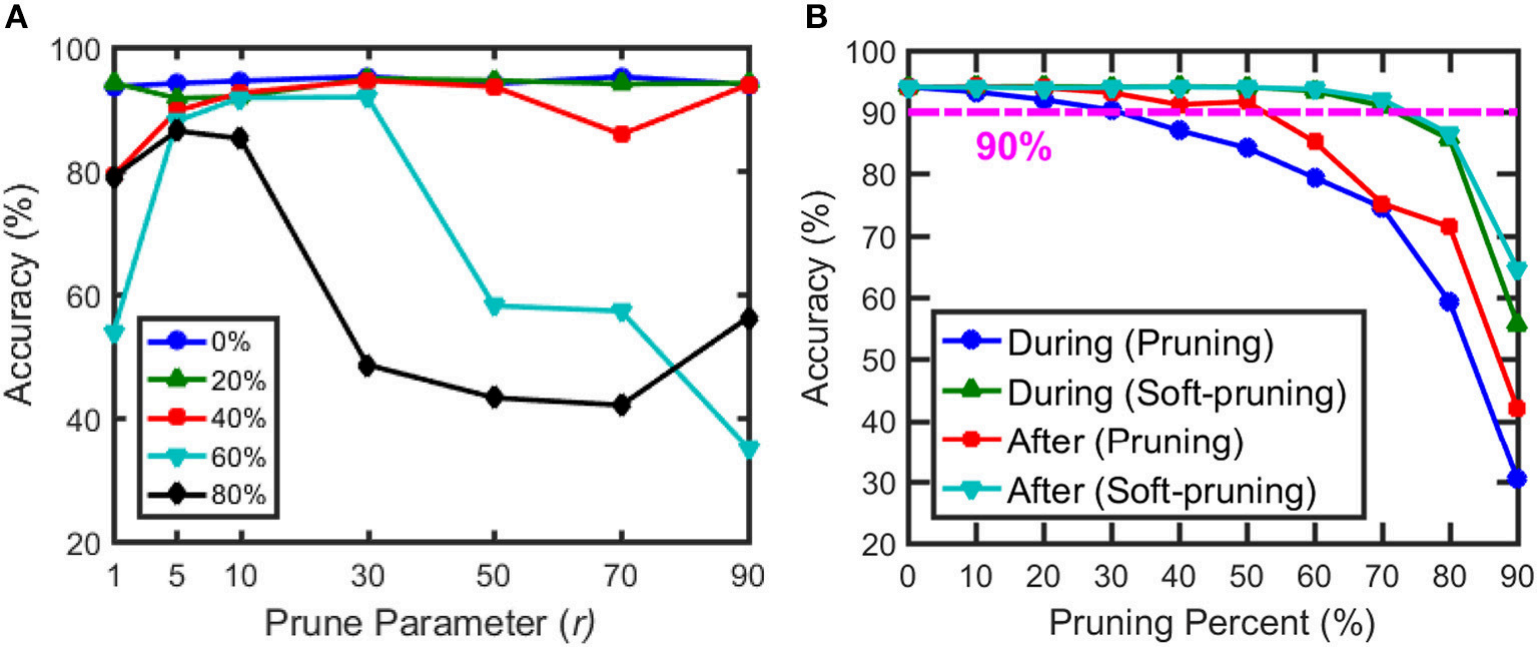
**(A)** Classification accuracy vs. prune parameter (*r*) for varying pruning percentages. Prune parameter is the criterion to decide when to prune for each neuron during training. **(B)** Classification accuracy vs. pruning percentage for pruning and soft-pruning when applied during training and after training. The data points are taken in steps of 10%. The dashed line represents classification accuracy of 90%. Soft-pruning during the training performs better than pruning especially for high pruning percentages. Soft-pruning maintains > 90% up to 75% pruning percentage while pruning falls below 90% at only 40% pruning. Although we focus on pruning during training, we also present results from pruning weights after training as a baseline for previously established pruning methods from the literature. The parameters used in the simulation are specified in Table 1.

Following the pruning methods described in section Pruning During Training, we investigate the performance through software simulations. Simulation of classification accuracy for different *p* values in Figure 9A suggests that *r* = 10 provides the high accuracy even for very large pruning percentages (up to 80%). Figure 9B shows the performance of pruning and soft-pruning for varying pruning percentages when applied after training and during training. When applied after training, pruning and soft-pruning are comparable with each other until ~50% pruning rate. After this point, the accuracy for the regular pruning method falls below ~90% at ~60% pruning rate, but with soft-pruning, the accuracy stays at ~90% until ~75% pruning rate. When each method is applied during training to save on computation of weight updates, the accuracy with pruning falls below ~90% at around a ~40% of pruning rate, and the accuracy with soft-pruning falls below this mark at a ~75% of pruning rate. The performance of pruning drops much earlier than soft-pruning because pruning compresses the representation of important weights and causes uneven firing between output neurons, as mentioned in section Pruning During Training. Soft-pruning during training provides comparable accuracy to pruning after training for up to 75% pruning rate while preventing excess computation on weight updates. Additionally, when soft-pruning is applied during training, the classification accuracy is maintained at ~94% with a pruning rate up to 60%. The aim of our work is mainly energy optimization during SNN training. Therefore, soft-pruning is chosen to maintain high accuracy with larger pruning percentage, while providing significant energy reduction during training. Since soft-pruning does not completely remove synaptic weights, it is not the best way to achieve memory optimization. Alternatively, conventional pruning (Han et al., 2015) presented in this work completely removes synaptic weights and it can be used to reduce the size of memory array used for inference with a little loss in accuracy (Figure 9B).

We also compare the number of weight updates of conventional STDP (Song et al., 2000), STDP used in this work and STDP used in this work with 50% soft-pruning in Table 2. Since conventional STDP demonstrated by Song et al. bound the number of weight update of excitatory synapses ($\bar{g_{a}}$) between 0 and $\bar{g_{\max}}$ while our STDP bound the weights between −1 and 1, the number of weight updates of conventional STDP and our STDP are almost the same as shown in the Table 2. On the other hand, STDP+Soft-pruning significantly reduces the number of device updates for 50% soft pruning. In addition, soft-pruning is conceptually similar to stop learning that has been proposed in semisupervised models (Brader et al., 2007; Mostafa et al., 2016). However, there are two major differences between soft-pruning and stop-learning. Our SNN training is unsupervised. Therefore, the criterion for our soft-pruning to stop updating the synapses is when an output neuron can generate enough count of consecutive spikes to a specific class of MNIST digits (See section Pruning during training in the manuscript). Brader et al. (2007) use a semi-supervised model. Therefore, stop-learning will happen when the total current *h* of an output neuron is in agreement with instructor signal (target). The threshold θ is chosen to determine if the output neuron satisfies the criterion. Furthermore, our soft-pruning stops updating part of the synapses of an output neuron depending on the pruning percentage the user set. This means that the un-pruned synapses still can be updated for the rest of the training. However, Brader et al. stop updating all the synapses of an output neuron once the stop-learning criterion is satisfied.

**Table 2:** The number of weight updates of conventional STDP (Song et al., 2000), STDP used in this work with and without 50% soft-pruning.

	**# of weight updates**
Conventional STDP (Song et al., 2000)	649289638
STDP (this work)	648669156
STDP (this work) + 50% Soft-pruning	357656929

Our classification accuracy is comparable to previous software implementations of unsupervised learning for the MNIST dataset with SNNs (Table 3). As can be seen from the table, multilayer SNNs (Diehl and Cook, 2015a; Kheradpisheh et al., 2017; Tavanaei and Maida, 2017; Ferré et al., 2018) generally have higher accuracy than single layer SNNs. However, the works with accuracy higher than 95% (Kheradpisheh et al., 2017; Tavanaei and Maida, 2017; Ferré et al., 2018) all require using multiple convolution and pooling layers, and other complex processing techniques, which are difficult to implement in hardware. Compared to the SNNs without convolution layers, our classification accuracy is much higher than previous single layer SNNs (Nessler et al., 2013; Al-Shedivat et al., 2015) and achieves performance very close to Diehl and Cook (2015a) with much fewer neurons and synapses. Our single layer SNN architecture does not require complex processing and is particularly suitable for easy hardware implementation. Differing from all previous approaches, we present a novel pruning method to reduce the number of updates to network parameters during SNN training. Hence, despite only part of the synapses in our network needing to be updated during training, our SNN still maintains a high classification accuracy with up a 75% pruning rate. Therefore, our pruning scheme can potentially reduce the energy consumption and training time in hardware implementation. The simple one-layer SNN architecture and STDP rule proposed in our work mainly focus on demonstrating the idea of pruning during the training. Scaling our SNN algorithm to larger datasets can be achieved by modifying the network architecture in several approaches such as by adding more fully connected layers (Diehl et al., 2015b; Lee et al., 2016; O'connor and Welling, 2016) or convolutional layers (Diehl et al., 2015b; Lee et al., 2016; Tavanaei and Maida, 2017; Kulkarni and Rajendran, 2018), adjusting learning rule and involving the supervision (Kulkarni and Rajendran, 2018).

**Table 3:** Classification accuracy comparison between this work and the state-of-the-art software demonstrations of unsupervised learning of SNNs on the MNIST dataset.

**Architecture**	**Complex Processing**	**Learning rule**	**#Neurons/synapses**	**Pruning during training**	**Performance**
Spiking deep neural network (Kheradpisheh et al., 2017)	Convolution, DOG filter, Pooling	Simplified STDP	N/A	None	98.4%
Multi layer (Ferré et al., 2018)	Convolution, Pooling, Dropout	Binary STDP	N/A	None	98.49%
Three layer (Tavanaei and Maida, 2017)	Convolution, Pooling	Probabilistic STDP	N/A	None	98.36%
Two layer (Diehl and Cook, 2015a)	None	Exponential STDP	7,184/5,017,600	None	95%
One layer (Al-Shedivat et al., 2015)	Population Coding	Probabilistic STDP	1,696/200,704	None	78.4%
One layer (Nessler et al., 2013)	Population Coding	Exponential STDP	808/70,800	None	80.14%
**One layer (this work)**	**None**	**Simplified STDP**	**898/~199,000**	**Yes (~75%)**	**94.05%**

*The table lists the complex processing techniques used, the learning rule, and the #Neurons/synapses used in each work. The table also indicates if pruning during training is involved in the work. The numbers of neurons are counted by summing the input and output neurons*.

**Figure 10:**
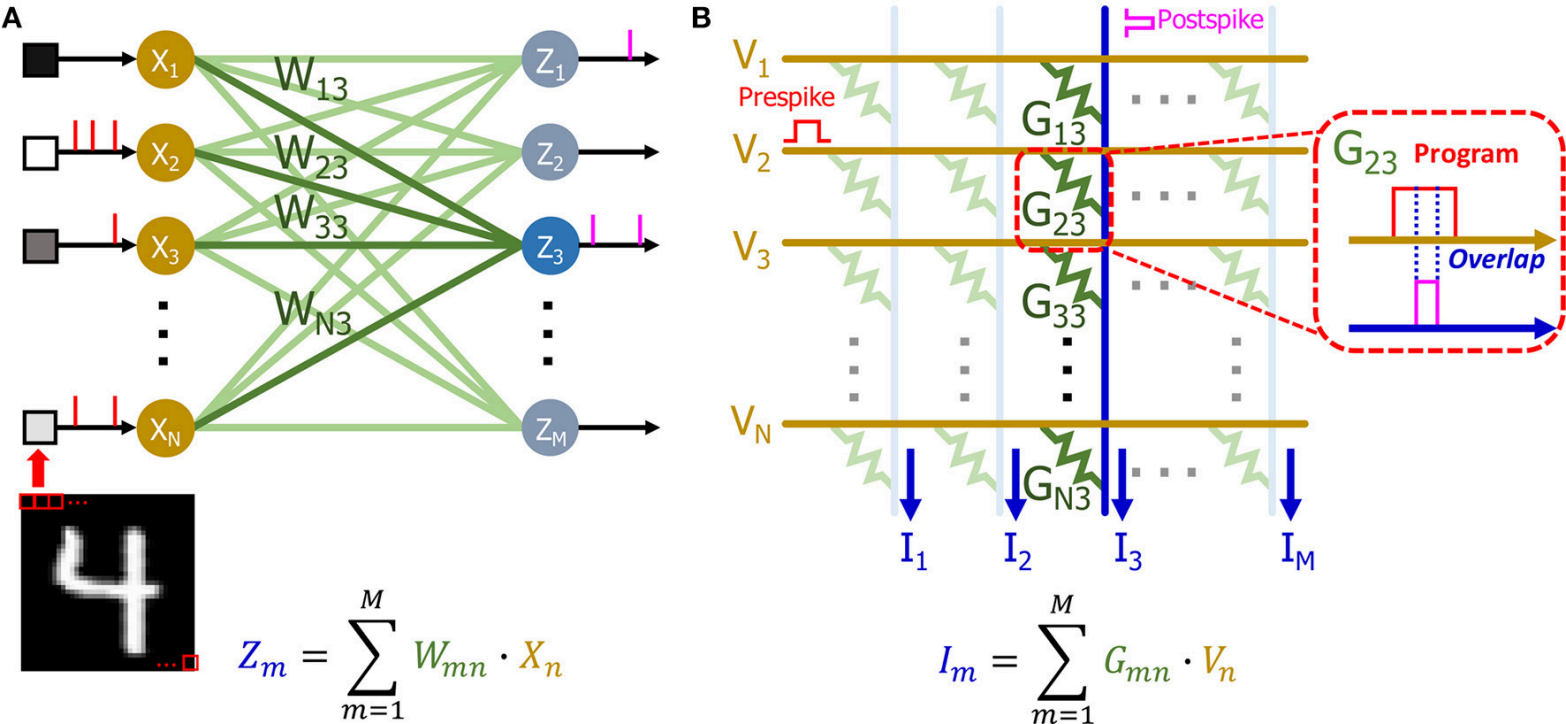
**(A)** Schematic of SNN with *n* input neurons and *m* output neurons. The pixel intensities of input image are decoded into passion spiking training and fed to the input of the network. The weights (*W*_13_, *W*_23_, …, *W*_*N*3_) of output neuron *Z*_3_ has been highlighted. **(B)** Schematic of a crossbar array based on eNVM devices. The input of **(A)** can be mapped to the voltage. The weights (*W*_13_, *W*_23_, …, *W*_*N*3_) are mapped to the conductance (*G*_13_, *G*_23_, …, *G*_*N*3_) of the devices. The weighted sum can be obtained by measuring the current at the end of each column. The postspike pulses are generated based on the weighted sums (*I*). The overlap of pre and post spike pulses as shown in callout window programs the device to different conductance states.

Our single layer SNN network (Figure 10A) can be directly mapped to a crossbar array based on eNVM devices (Figure 10B) to perform online learning. The input of the network is decoded into a Poisson spike train based on the pixel intensity (see section Input layer for details) and it can be mapped to the input voltage spikes of the crossbar array (Figure 10A). There are many demonstrations showing that eNVM devices can have multilevel conductance states to emulate analog weight tuning (Jo et al., 2010; Kuzum et al., 2011). Therefore, the weights in the SNN can be represented using the conductance of eNVM devices. Since the weights in our network is ranging from −1 to 1, there are two ways to use device conductance to represent the weights. One approach could be using a single device to represent a synaptic weight. The weights in the network are linearly transformed to the conductance range as shown in Equation (6) for the hardware implementation (Serb et al., 2016; Kim et al., 2018; Li et al., 2018; Oh et al., 2018; Shi et al., 2018).

(6) $$G=\;W\frac{\left(G_{\max\;-\;}G_{\min}\right)}{2}+\;\frac{\left(G_{\max+}G_{\min}\right)}{2}$$

An alternative approach could be using of two devices as one synaptic weight as shown in previous literature (Burr et al., 2015; Li et al., 2018). Both positive and negative weights can be represented by taking the difference between conductance of two devices (*G* = *G*^+^− *G*^−^). The weighted sum operation for calculating membrane potential (see section Output layer for details) can be calculated in a single step by accumulating the current flowing through each column in the crossbar array (Eryilmaz et al., 2016). Our STDP weight update rule can be realized by overlapping of the prespike and postspike pulses (Figure 10B) to program the device to different conductance levels, as shown in previous demonstrations (Kuzum et al., 2011, 2012).

In order to implement pruning in hardware, the pruned cells need to be flagged to prevent them from being updated further. One solution is to use an extra binary device associated with each eNVM synaptic weight to serve as a hardware pruning flag. This binary device is initially programmed to “0” (the lowest conductance state), to indicate that the cell has not been pruned. We update the pruning flag of an output neuron's weights to “1” (the highest conductance state) when it has been pruned during training. Before the weight update, we read the hardware flag of the winning neuron's weight to decide whether or not to update. The weights are only pruned once during the entire training. As a result, each hardware flag is just written once and hence the energy overhead will be negligible. However, the hardware pruning flag will slightly increase the area of the array. If the size of the array is crucial for a system, an alternative way can be used to implement the hardware flag without area overhead. The pruned cells can be reset to a very low conductance state with additional reset current (Arita et al., 2015; Xia et al., 2017). Such cells generally require reforming to be programmed to a multi-level conductance state regime again (Wong et al., 2012). Therefore, the pruned cells will not be further updated during training and we can use its very low conductance state as pruning flag.

**Figure 11:**
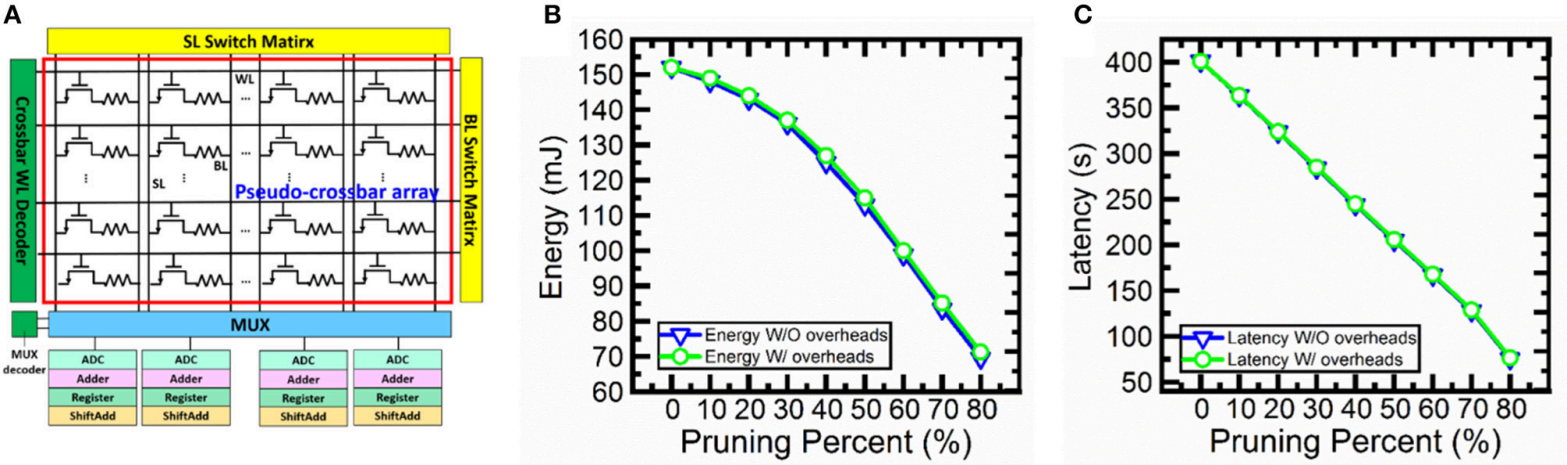
**(A)** Analog synaptic core uses a single cell with multi-level conductance states to represent one synaptic weight. One transistor is added to each cell in order to avoid sneak path problem. The crossbar wordline (WL) decoder can activate all WLs, bitline (BL) read out the weighted sum results, and source line (SL) can be used to perform weight update. Multiplexer (MUX) is used to share the neuron circuitry. The neuron circuit contains analog-to-digital converters (ADCs), adders, registers and shift adders, which are used to perform weighted sum. **(B)** The energy and **(C)** latency without and with overhead estimation for soft-pruning from 10 to 80% with a step of 10% using SNN+NeuroSim. Without overheads (W/O overheads) results mean that flagging mechanism is implemented in software. With overheads (W/ overheads) results mean that flagging mechanism is implemented in hardware.

In order to confirm the feasibility of the proposed hardware implementation of pruning during SNN training. We perform circuit-level benchmarking simulations with NeuroSim (Chen et al., 2018) to evaluate the performance of a full system of analog synaptic core as shown in Figure 11A. NeuroSim is a C++ based simulator with hierarchical organization starting from experimental device data and extending to array architectures with peripheral circuit modules and algorithm-level neural network models (Chen et al., 2018). We develop a SNN platform for NeuroSim (SNN+NeuroSim). SNN+NeuroSim can simulate circuit-level performance metrics (area, energy and latency) at run-time of online learning using eNVM arrays. We implement the hardware flagging mechanism of pruning in SNN+NeuroSim and estimate energy and latency overheads caused by flagging mechanism. Figures 11B,C show energy and latency without and with overheads due to pruning. The results show that the energy and latency can be significantly decreased as the pruning percentages increase. The results also suggest that energy consumption and latency do not significantly increase due to the overheads associated with the hardware flag for the pruning percentages from 10 to 80%.

## Conclusion

In this work, we first demonstrate a low-complexity single layer SNN training model for unsupervised learning on MNIST. We then develop a new method to prune during training for SNNs. Our pruning scheme exploits the output spike firing of the SNN to reduce the number of weight updates during network training. With this method, we investigate the impact of pruning and soft-pruning on classification accuracy. We show that our SNN can maintain high classification accuracy (~90%) on the MNIST dataset and the network can be extensively pruned (75% pruning rate) during training. We also discuss and simulate the possible hardware implementation of our SNN and pruning algorithm with eNVM crossbar arrays using SNN+NeuroSim. Our algorithmic optimization approach can be applied to improve network level energy efficiency of other SNNs with eNVM arrays for in-memory computing applications, enabling online learning of SNNs in power-limited settings.

## Author Contributions

YS, LN, and DK conceived the idea. YS and LN developed the pruning algorithm. YS, LN, and SO implemented unsupervised learning neural network simulation, and analysis the data obtained from the simulation. All authors wrote the manuscript, discussed the results and commented on the manuscript. DK supervised the work.

### Conflict of Interest Statement

The authors declare that the research was conducted in the absence of any commercial or financial relationships that could be construed as a potential conflict of interest.

## References

[B1] Alibart F. Zamanidoost E. Strukov D. B. (2013). Pattern classification by memristive crossbar circuits using ex situ and in situ training. Nat. Commun. 4:2072. 10.1038/ncomms3072 23797631

[B2] Al-Shedivat M. Naous R. Cauwenberghs G. Salama K. N. (2015). Memristors empower spiking neurons with stochasticity. IEEE J. Emerg. Select. Topics Circuits Syst. 5, 242–253. 10.1109/JETCAS.2015.2435512

[B3] Ankit A. Sengupta A. Panda P. Roy K. (2017). Resparc: a reconfigurable and energy-efficient architecture with memristive crossbars for deep spiking neural networks, in Proceedings of the 54th Annual Design Automation Conference 2017: ACM (Austin, TX), 27.

[B4] Arita M. Takahashi A. Ohno Y. Nakane A. Tsurumaki-Fukuchi A. Takahashi Y. (2015). Switching operation and degradation of resistive random access memory composed of tungsten oxide and copper investigated using in-situ TEM. Sci. Rep. 5:17103. 10.1038/srep17103 26611856 PMC4661473

[B5] Brader J. M. Senn W. Fusi S. (2007). Learning real-world stimuli in a neural network with spike-driven synaptic dynamics. Neural Comput. 19, 2881–2912. 10.1162/neco.2007.19.11.2881 17883345

[B6] Burr G. W. Shelby R. M. Sidler S. Di Nolfo C. Jang J. Boybat I. (2015). Experimental demonstration and tolerancing of a large-scale neural network (165 000 synapses) using phase-change memory as the synaptic weight element. IEEE Trans. Electron Devices 62, 3498–3507. 10.1109/TED.2015.2439635

[B7] Cao Y. Chen Y. Khosla D. (2015). Spiking deep convolutional neural networks for energy-efficient object recognition. Int. J. Comput. Vis. 113, 54–66. 10.1007/s11263-014-0788-3

[B8] Chen P.-Y. Peng X. Yu S. (2018). NeuroSim: a circuit-level macro model for benchmarking neuro-inspired architectures in online learning. IEEE Trans. Comput. Aided Design Integrat. Circuits Syst. 37, 3067–3080. 10.1109/TCAD.2018.2789723

[B9] Choi S. Sheridan P. Lu W. D. (2015). Data clustering using memristor networks. Sci. Rep. 5:10492. 10.1038/srep10492 26020412 PMC4650685

[B10] Cruz-Albrecht J. M. Yung M. W. Srinivasa N. (2012). Energy-efficient neuron, synapse and STDP integrated circuits. IEEE Trans. Biomed. Circuits Syst. 6, 246–256. 10.1109/TBCAS.2011.2174152 23853146

[B11] Deger M. Helias M. Rotter S. Diesmann M. (2012). Spike-timing dependence of structural plasticity explains cooperative synapse formation in the neocortex. PLoS Comput. Biol. 8:e1002689. 10.1371/journal.pcbi.1002689 23028287 PMC3447982

[B12] Deger M. Seeholzer A. Gerstner W. (2017). Multicontact co-operativity in spike-timing-dependent structural plasticity stabilizes networks. Cerebral. Cortex 28, 1396–1415. 10.1093/cercor/bhx339 29300903 PMC6041941

[B13] Diehl P. U. Cook M. (2015a). Unsupervised learning of digit recognition using spike-timing-dependent plasticity. Front. Comput. Neurosci. 9:99. 10.3389/fncom.2015.00099 26941637 PMC4522567

[B14] Diehl P. U. Neil D. Binas J. Cook M Liu S.-C. Pfeiffer M. (2015b). Fastclassifying, high-accuracy spiking deep networks through weight and threshold balancing, in 2015 International Joint Conference on Neural Networks (IJCNN): IEEE (Killarney), 1–8.

[B15] Eryilmaz S. B. Neftci E. Joshi S. Kim S. Brightsky M. Lung H.-L. (2016). Training a probabilistic graphical model with resistive switching electronic synapses. IEEE Trans. Electron Devices 63, 5004–5011. 10.1109/TED.2016.2616483

[B16] Ferré P. Mamalet F. Thorpe S. J. (2018). Unsupervised feature learning with winner-takes-all based STDP. Front. Comput. Neurosci. 12:24. 10.3389/fncom.2018.00024 29674961 PMC5895733

[B17] Ge R. Wu X. Kim M. Shi J. Sonde S. Tao L.. (2017). Atomristor: nonvolatile resistance switching in atomic sheets of transition metal dichalcogenides. Nano Lett. 18, 434–441. 10.1021/acs.nanolett.7b04342 29236504

[B18] Gupta A. Long L. N. (2007). Character recognition using spiking neural networks, in Neural Networks, 2007. IJCNN 2007. International Joint Conference on: IEEE (Orlando, FL), 53–58.

[B19] Han S. Pool J. Tran J. Dally W. (2015). Learning both weights and connections for efficient neural network, in Advances in Neural Information Processing Systems (Montreal, QC), 1135–1143.

[B20] Iglesias J. Villa A. E. (2007). Effect of stimulus-driven pruning on the detection of spatiotemporal patterns of activity in large neural networks. BioSystems 89, 287–293. 10.1016/j.biosystems.2006.05.020 17324499

[B21] Jo S. H. Chang T. Ebong I. Bhadviya B. B. Mazumder P. Lu W. (2010). Nanoscale memristor device as synapse in neuromorphic systems. Nano Lett. 10, 1297–1301. 10.1021/nl904092h 20192230

[B22] Kappel D. Habenschuss S. Legenstein R. Maass W. (2015). Synaptic sampling: a bayesian approach to neural network plasticity and rewiring, in Advances in Neural Information Processing Systems (Montreal, QC), 370–378.

[B23] Kheradpisheh S. R. Ganjtabesh M. Thorpe S. J. Masquelier T. (2017). STDP-based spiking deep convolutional neural networks for object recognition. Neural Netw. (2017). 10.1016/j.neunet.2017.12.005 29328958

[B24] Kijsirikul B. Chongkasemwongse K. (2001). Decision tree pruning using backpropagation neural networks, in Proceedings of IEEE International Conference on Neural Networks (Washington, DC), 1876–1880.

[B25] Kim H. Kim T. Kim J. Kim J.-J. (2018). Deep neural network optimized to resistive memory with nonlinear current-voltage characteristics. ACM J. Emerg. Technol. Comput. Syst. (JETC) 14:15 10.1145/3145478

[B26] Kulkarni S. R. Rajendran B. (2018). Spiking neural networks for handwritten digit recognition-Supervised learning and network optimization. Neural Networks 103, 118–127. 10.1016/j.neunet.2018.03.019 29674234

[B27] Kuzum D. Jeyasingh R. G. Lee B. Wong H.-S. P. (2011). Nanoelectronic programmable synapses based on phase change materials for brain-inspired computing. Nano Lett. 12, 2179–2186. 10.1021/nl201040y 21668029

[B28] Kuzum D. Jeyasingh R. G. D. Yu S. Wong H. S. P. (2012). Low-energy robust neuromorphic computation using synaptic devices. IEEE Trans. Electron Devices 59, 3489–3494. 10.1109/TED.2012.2217146

[B29] Lee J. H. Delbruck T. Pfeiffer M. (2016). Training deep spiking neural networks using backpropagation. Front. Neurosci. 10:508. 10.3389/fnins.2016.00508 27877107 PMC5099523

[B30] Li C. Hu M. Li Y. Jiang H. Ge N. Montgomery E. (2018). Analogue signal and image processing with large memristor crossbars. Nat. Electron. 1, 52–59. 10.1038/s41928-017-0002-z

[B31] Maass W. (1997). Networks of spiking neurons: the third generation of neural network models. Neural Networks 10, 1659–1671. 10.1016/S0893-6080(97)00011-7

[B32] Merolla P. A. Arthur J. V. Alvarez-Icaza R. Cassidy A. S. Sawada J. Akopyan F.. (2014). A million spiking-neuron integrated circuit with a scalable communication network and interface. Science 345, 668–673. 10.1126/science.1254642 25104385

[B33] Mnih V. Kavukcuoglu K. Silver D. Rusu A. A. Veness J. Bellemare M. G.. (2015). Human-level control through deep reinforcement learning. Nature 518, 529–533. 10.1038/nature14236 25719670

[B34] Mostafa H. Mayr C. Indiveri G. (2016). Beyond spike-timing dependent plasticity in memristor crossbar arrays, in 2016 IEEE International Symposium on Circuits and Systems (ISCAS): IEEE, 926–929.

[B35] Neftci E. Das S. Pedroni B. Kreutz-Delgado K. Cauwenberghs G. (2014). Event-driven contrastive divergence for spiking neuromorphic systems. Front. Neurosci. 7:272. 10.3389/fnins.2013.00272 24574952 PMC3922083

[B36] Nessler B. Pfeiffer M. Buesing L. Maass W. (2013). Bayesian computation emerges in generic cortical microcircuits through spike-timing-dependent plasticity. PLoS Comput. Biol. 9:e1003037. 10.1371/journal.pcbi.1003037 23633941 PMC3636028

[B37] O'connor P. Welling M. (2016). Deep spiking networks. arXiv preprint arXiv:1602.08323.

[B38] Oh S. Shi Y. Liu X. Song J. Kuzum D. (2018). Drift-enhanced unsupervised learning of handwritten digits in spiking neural network with PCM synapses. IEEE Electron Device Lett. 39, 1768–1771. 10.1109/LED.2018.2872434

[B39] Panda P. Srinivasan G. Roy K. (2017a). Convolutional spike timing dependent plasticity based feature learning in spiking neural networks. arXiv preprint arXiv:1703.03854.

[B40] Panda P. Srinivasan G. Roy K. (2017b). EnsembleSNN: distributed assistive STDP learning for energy-efficient recognition in spiking neural networks, in 2017 International Joint Conference on Neural Networks (IJCNN): IEEE, 2629–2635.

[B41] Perfors A. Tenenbaum J. B. Griffiths T. L. Xu F. (2011). A tutorial introduction to bayesian models of cognitive development. Cognition 120, 302–321. 10.1016/j.cognition.2010.11.015 21269608

[B42] Prezioso M. Merrikh-Bayat F. Hoskins B. Adam G. Likharev K. K. Strukov D. B. (2015). Training and operation of an integrated neuromorphic network based on metal-oxide memristors. Nature 521:61–64. 10.1038/nature14441 25951284

[B43] Rathi N. Panda P. Roy K. (2018). STDP based pruning of connections and weight quantization in spiking neural networks for energy-efficient recognition. IEEE Trans. Comput. Aided Design Integrat. Circuits Syst. 38, 668–677. 10.1109/TCAD.2018.2819366

[B44] Sengupta A. Parsa M. Han B. Roy K. (2016). Probabilistic deep spiking neural systems enabled by magnetic tunnel junction. IEEE Trans. Electr. Devices 63, 2963–2970. 10.1109/TED.2016.2568762

[B45] Serb A. Bill J. Khiat A. Berdan R. Legenstein R. Prodromakis T. (2016). Unsupervised learning in probabilistic neural networks with multi-state metal-oxide memristive synapses. Nat. Commun. 7:12611. 10.1038/ncomms12611 27681181 PMC5056401

[B46] Shi Y. Nguyen L. Oh S. Liu X. Koushan F. Jameson J. R.. (2018). Neuroinspired unsupervised learning and pruning with subquantum CBRAM arrays. Nat. Commun. 9:5312. 10.1038/s41467-018-07682-0 30552329 PMC6294253

[B47] Song S. Miller K. D. Abbott L. F. (2000). Competitive Hebbian learning through spike-timing-dependent synaptic plasticity. Nat. Neurosci. 3, 919–926. 10.1038/78829 10966623

[B48] Spiess R. George R. Cook M. Diehl P. U. (2016). Structural plasticity denoises responses and improves learning speed. Front. Comput. Neurosci. 10:93. 10.3389/fncom.2016.00093 27660610 PMC5014863

[B49] Srinivasan G. Sengupta A. Roy K. (2016). Magnetic tunnel junction based long-term short-term stochastic synapse for a spiking neural network with on-chip STDP learning. Sci. Rep. 6:29545. 10.1038/srep29545 27405788 PMC4942786

[B50] Tavanaei A. Maida A. S. (2017). Multi-layer unsupervised learning in a spiking convolutional neural network, in Neural Networks (IJCNN), International Joint Conference on: IEEE (Anchorage, AK), 2023–2030.

[B51] Tavanaei A. Masquelier T. Maida A. S. (2016). Acquisition of visual features through probabilistic spike-timing-dependent plasticity, in Neural Networks (IJCNN), 2016 International Joint Conference on: IEEE (Vancouver, BC), 307–314.

[B52] Vincent A. F. Larroque J. Zhao W. Romdhane N. B. Bichler O. Gamrat C.. (2014). Spin-transfer torque magnetic memory as a stochastic memristive synapse, in 2014 IEEE International Symposium on Circuits and Systems (ISCAS): IEEE (Melbourne, VIC), 1074–1077. 10.1109/TBCAS.2015.2414423 25879967

[B53] Wong H.-S. P. (2018). The end of the road for 2 Dscaling of silicon CMOS and the future of device technology, in 2018 76th Device Research Conference (DRC): IEEE (Santa Barbara, CA), 1–2.

[B54] Wong H.-S. P. Lee H.-Y. Yu S. Chen Y.-S. Wu Y. Chen P.-S. (2012). Metal-oxide RRAM. Proc. IEEE. 100, 1951–1970. 10.1109/JPROC.2012.2190369

[B55] Xia L. Liu M. Ning X. Chakrabarty K. Wang Y. (2017). Fault-tolerant training with on-line fault detection for RRAM-based neural computing systems, in Proceedings of the 54th Annual Design Automation Conference 2017: ACM, 33.

